# C-Reactive Protein (CRP) Gene Polymorphisms, CRP Levels, and Risk of Incident Coronary Heart Disease in Two Nested Case-Control Studies

**DOI:** 10.1371/journal.pone.0001395

**Published:** 2008-01-02

**Authors:** Jennifer K. Pai, Kenneth J. Mukamal, Kathryn M. Rexrode, Eric B. Rimm

**Affiliations:** 1 Department of Epidemiology, Harvard School of Public Health, Boston, Massachusetts, United States of America; 2 Channing Laboratory, Department of Medicine, Brigham and Women's Hospital, Harvard Medical School, Boston, Massachusetts, United States of America; 3 Division of General Medicine and Primary Care, Beth Israel Deaconess Medical Center, Boston, Massachusetts, United States of America; 4 Division of Preventive Medicine, Department of Medicine, Brigham and Women's Hospital, Harvard Medical School, Boston, Massachusetts, United States of America; Innsbruck Medical University, Austria

## Abstract

**Background:**

C-reactive protein (CRP), an acute phase reactant and marker of inflammation, has been shown to predict risk of incident cardiovascular events. However, few studies have comprehensively examined six common single-nucleotide polymorphisms (SNPs) in the CRP gene, haplotypes, and plasma CRP levels with risk of coronary heart disease (CHD).

**Methods and Findings:**

We conducted parallel nested case-control studies within two ongoing, prospective cohort studies of U.S. women (Nurses' Health Study) and men (Health Professionals Follow-up Study). Blood samples were available in a subset of 32,826 women and 18,225 men for biomarker and DNA analyses. During 8 and 6 years of follow-up, 249 women and 266 men developed incident nonfatal myocardial infarction or fatal CHD, and controls (498 women, 531 men) were matched 2:1 on age, smoking, and date of blood draw from participants free of cardiovascular disease at the time the case was diagnosed. Among both women and men, minor alleles were significantly associated with higher CRP levels for SNPs 1919A>T and 4741G>C, but associated with lower CRP levels for SNPs 2667G>C and 3872C>T. SNP 2667G>C was individually associated with increased risk of CHD in both women [OR 1.57 (95% CI 1.01–2.44); p = 0.047] and men [1.93 (95% CI 1.30–2.88); p = 0.001]. Two of the five common haplotypes were associated with lower CRP levels, and Haplotype 4 which included minor alleles for 2667 and 3872 was associated with significantly lower CRP levels and an elevated risk of CHD. The remaining SNPs or haplotypes were not associated with CHD in both populations.

**Conclusions:**

Common variation in the CRP gene was significantly associated with plasma CRP levels; however, the association between common SNPs and CRP levels did not correspond to a predicted change in CHD risk. The underlying inflammatory processes which predict coronary events cannot be captured solely by variation in the CRP gene.

## Introduction

Inflammatory processes are involved in the initiation and progression of atherosclerotic lesions, as well as in the development of atheroma complications,[1] and markers of inflammation may reflect subclinical vascular inflammation and be useful diagnostic tools.[2] C-reactive protein (CRP), an acute phase reactant, is a marker of inflammation, and has been shown to independently predict risk of future cardiovascular events among initially healthy men and women.[3]–[6] However, since CRP levels can be influenced by age, smoking, body mass index, and other clinical or environmental risk factors,[7]–[9] it remains to be determined whether CRP acts causally in atherogenesis, in addition to serving as a reliable marker of future events.[10]

Recent family studies suggest heritability estimates of CRP ranging from 27–40%,[11], [12] and it is hypothesized that genetic variation in the CRP gene may influence plasma CRP levels and subsequent risk of CHD. Several studies have reported individual single nucleotide polymorphisms (SNPs) to be associated with CRP levels,[13]–[21] and of these, Zee et al.,[15] which included SNP 2667G>C, Casas et al.,[19] which included SNP 3014G>A, also examined associations with cardiovascular events. Both studies reported no association with the individual SNPs and risk of cardiovascular disease.

To date, few studies of both men and women have simultaneously examined common genetic variation in the CRP gene, haplotypes, plasma CRP levels, and risk of incident cardiovascular events. In the Rotterdam Study, the common haplotypes associated with higher CRP levels were not associated with risk of coronary heart disease.[22] Similarly, among men in the Physicians' Health Study, none of the common SNPs associated with higher CRP levels were associated with an increased risk of atherothrombotic events, whereas one SNP not associated with CRP was associated with a decreased risk of myocardial infarction.[23] Alternatively, in the Cardiovascular Health Study among elderly adults, haplotypes were significantly associated with CRP levels, and stroke and cardiovascular mortality, but not associated with myocardial infarction.[24] Because these studies included some, but not all of the same SNPs, estimated haplotypes are not directly comparable, and thus, summary conclusions cannot be made. Utilizing the existing sequencing data and available haplotype-tagging SNPs, we set out to comprehensively examine common variation in the CRP gene with plasma CRP levels and risk of incident coronary heart disease among two independent populations of middle-aged men and women.

## Methods

### Study Population

The Nurses' Health Study (NHS) and the Health Professionals Follow-up Study (HPFS) are prospective cohort investigations among 121,700 female U.S. registered nurses aged 30–55 years at baseline in 1976 (NHS) and 51,529 U.S. male health professionals, aged 40–75 years at baseline in 1986 (HPFS). Information about health and disease is assessed biennially and information about diet every four years by self-administered questionnaires.[25], [26] Between 1989 and 1990, a blood sample was requested from all participants of the NHS, and returned from 32,826 women. Similarly, between 1993 and 1995, a blood sample was returned from 18,225 men in the HPFS. Participants who provided blood samples were similar in terms of cardiovascular risk factors to those who did not, albeit somewhat younger. In the NHS, among women without cardiovascular disease or cancer prior to blood draw, we identified 249 women with incident nonfatal myocardial infarction (MI) or fatal coronary heart disease (CHD) over 8 years of follow-up. In the HPFS, we similarly identified 266 men with incident nonfatal MI or fatal CHD over 6 years of follow-up. Using risk-set sampling which selects controls from the base population that gave rise to the cases,[27] controls were randomly selected 2:1 matched on age, smoking, date of blood draw, and fasting status (women only) from participants free of cardiovascular disease at the time the case was diagnosed.

The study protocol was approved by the Institutional Review Board of the Brigham and Women's Hospital and the Harvard School of Public Health Human Subjects Committee Review Board.

### Assessment of coronary heart disease

Myocardial infarction was confirmed by study physicians blinded to participant's exposure status if it met the World Health Organization's criteria (symptoms plus either diagnostic electrocardiographic changes or elevated levels of cardiac enzymes).[28], [29] Deaths were identified from state vital records and the National Death Index or reported by the participant's next of kin or the postal system. Fatal coronary heart disease was confirmed by hospital records or on autopsy, or if coronary heart disease was listed as the cause of death on the death certificate, if it was the underlying and most plausible cause, and if evidence of previous coronary heart disease was available. Among women, 80.3% of the cases were nonfatal MI, and 19.7% were fatal CHD. Among men, 73.7% of the cases were nonfatal MI, and 26.3% were fatal CHD.

### Measurement of biochemical variables

Blood samples were collected in sodium heparin tubes for women and liquid EDTA for men, placed on ice packs, stored in Styrofoam containers, returned to our laboratory via overnight courier, and centrifuged and aliquoted for storage in liquid nitrogen freezers (−130°C or colder). Inflammatory marker levels were largely unaffected by transport conditions and reproducible within persons over time.[30], [31]

Plasma biomarkers were measured for nonfatal MI and fatal CHD cases and their controls. C-reactive protein concentrations were determined using an immunoturbidimetric high sensitivity assay using reagents and calibrators from Denka Seiken (Niigata, Japan) with assay day-to-day variability between 1 and 2%. This assay has a sensitivity of 0.1 mg/L, and the day-to-day variability of the assay at concentrations of 0.91, 3.07, and 13.38 mg/L were 2.8, 1.6, and 1.1%, respectively. The intra-assay coefficients of variation were <10% for both HPFS and NHS. Total, HDL, and directly assessed LDL cholesterol, and triglycerides were measured using standard methods with reagents from Roche Diagnostics (Indianapolis, IN) and Genzyme (Cambridge, MA). Study samples were sent to the laboratory for analysis in randomly ordered batches, and the laboratory was blinded to case-control status.

### SNP selection and genotyping

To comprehensively assess the genetic variation in the CRP gene, we utilized a multi-stage approach. (1.) First, Carlson et al.[32] had resequenced the CRP gene in 23 unrelated European-descent individuals (EA) in the Utah/Centre d'Etude du Polymorphisme Humain (CEPH) panel (Coriell Institute for Medical Research, Camden, NJ), and using linkage disequilibrium had selected tagSNPs that summarized the common variation in the CRP gene. The five tagSNPs polymorphic in EAs, included a triallelic 1440C>T>A (rs3091244) and a commonly studied rare 2667G>C. (2.) Additionally, we conducted a comprehensive search of SNPs available in dbSNP (as of August 2005) which were polymorphic in Caucasians and had a minor allele frequency >1%, and resulted in two additional SNPs with low minor allele frequency (MAF) [4362G>C and 5606G>T]. (3.) Finally, the International HapMap Project[33] showed that two tagSNPs [1919A>T and 3872C>T] were sufficient to assess the variation in the gene, and these two were already included in the selected SNPs. The triallelic SNP could not be genotyped using Taqman; however, previous LD analyses[9] showed that SNPs 3872C>T and 5237A>G together serve as a proxy so these two SNPs were used instead.

DNA was extracted from the buffy coat fraction of centrifuged blood using the QIAmp Blood Kit (Qiagen, Chatsworth, CA). The primary genotyping technique was Taqman SNP allelic discrimination by means of an ABI 7900HT (Applied Biosystems, Foster City, CA) and the primers and probes used were designed by Applied Biosystems: 1919A>T (rs1417938), 2667G>C (rs1800947), 3014G>A (rs1130864), 3872C>T (rs1205), 4362A>T (rs3093080), 4741G>C (rs3093068), 5237A>G (rs2808630), and 5606G>T (rs3093071). Replicate quality control samples were included and genotyped with 100% concordance. Cases were not perfectly matched to controls because a few subjects could not be genotyped with this platform, but genotype call rates did not differ between cases and controls. We initially genotyped the NHS, and then conducted a replication study in the HPFS. Among NHS, the minor allele frequencies for 4362A>T (0.6% cases, 1.1% controls) and 5606G>T (1% cases, 0.5% controls) were very low; thus, we did not further genotype them in the HPFS. The remaining 6 SNPs were selected for final analyses.

### Statistical analyses

All analyses were conducted separately for men and women. For baseline characteristics, we used ANOVA methods to compare least square means adjusted for matching factors, and the Chi-square and/or Fisher's Exact tests to compare proportions between cases and controls, between genotype and allele frequencies, and to assess Hardy-Weinberg equilibrium. The primary genetic model was additive (genotype as a continuous variable). Conditional and unconditional logistic regression adjusting for matching factors and multivariate covariates were used to estimate the relative risk (RR) and results were similar. With risk-set sampling, the odds ratio derived from the logistic regression directly estimates the hazard ratio, and thus, the relative risk.[27] In our multivariable model we further adjusted for alcohol intake (nondrinker, 0.1 to 4.9 g/d, 5.0 to 14.9 g/d, 15.0 to 29.9 g/d, ≥30.0 g/d), body mass index (<25, 25 to 29.9, ≥30 kg/m^2^), physical activity (quintiles), nonsteroidal anti-inflammatory drug (NSAID) use (yes/no), parental history of CHD before the age of 60 (yes/no), history of diabetes (yes/no) and hypertension (yes/no) at baseline, total:HDL-cholesterol ratio (quintiles), and hormone therapy among women (never, past, current). Baseline was defined as the year of blood draw. Lewontin's D prime (D') and the correlation coefficient (r^2^) were calculated as two measures of linkage disequilibrium (LD) between CRP polymorphisms among control participants. CRP levels were not normally distributed and were log-transformed for analyses. Multivariable linear regression analyses were conducted to model the association between CRP SNPs and CRP levels among control participants.

Using an expectation-maximization algorithm in SAS Genetics, we estimated frequencies for the five most common (>5% frequency) haplotypes in each population and imputed subject-specific expected haplotypes.[34], [35] Haplotype-specific odds ratios based on the additive model were calculated using unconditional logistic regression adjusting for matching factors with the most frequent haplotype set as the reference. The likelihood ratio test was used to calculate the global p-value comparing the model with haplotypes to the model without haplotypes. We present otherwise uncorrected p-values, and emphasize the 95% confidence intervals around risk estimates from two independent populations.

All p-values presented are two-tailed and p-values below 0.05 were considered statistically significant. All analyses were performed using SAS version 9 (SAS Institute, Cary, NC).

## Results

### Baseline characteristics

Cardiovascular risk factors were higher or more frequent among the cases compared to the controls in both men and women (**Table 1**), and for most of the CRP genotype frequencies, there were no statistical differences by case and control status (**Table 2**). The 2667C allele was significantly more frequent among cases than controls in women (p = 0.05) and men (0.006), and the 5237G allele was more frequent among cases then controls in women only (p = 0.004). The observed allele frequencies for all SNPs, except for 5237A>G among women (p = 0.03) and 4741G>C among men (p = 0.001), were within Hardy-Weinberg equilibrium among the controls. The pairwise LD measures and correlation coefficients between CRP polymorphisms were analyzed among the control women and men (**Table 3**). The minor alleles for 1919A>T and 3014G>A were in high pairwise LD (r^2^>0.95), whereas 2667G>C, 3872C>T, 4741G>C, and 5237A>G were not in high LD (r^2^<0.30).

**Table 1:** Baseline characteristics of participants with incident CHD (cases) and matched* controls among women (Nurses' Health Study) and men (Health Professionals Follow-up Study)†

	Women			Men		
Characteristics	Cases	Controls	P‡	Cases	Controls	P‡
N	249	498		266	531	
Age (years)	60.3 ± 0.09	60.3 ± 0.06	0.93	65.2 ± 0.13	65.1 ± 0.09	0.93
Current Smokers (%)	32.1	31.9	-	12.7	12.7	-
Caucasian§ (%)	96.1	96.5	0.83	98.1	98.4	>0.99
Postmenopausal (%)	90.3	87.8	0.32	-	-	-
Body mass index (kg/m^2^)	26.9 ± 0.31	25.4 ± 0.22	<0.001	26.2 ± 0.21	25.7 ± 0.15	0.05
History of hypertension (%)	57.4	29.3	<0.001	42.1	30.9	0.002
History of diabetes (%)	19.7	6.6	<0.001	9.4	4.5	0.007
Parental history of CHD before age 60 (%)	27.7	12.3	<0.001	15.0	10.9	0.10
Total:HDL-cholesterol ratio	4.88 ± 0.09	4.05 ± 0.06	<0.001	5.37 ± 0.09	4.75 ± 0.06	<0.001
CRP (mg/L)	3.08 (2.67, 3.55)	2.16 (1.95, 2.39)	<0.001	1.58 (1.38, 1.80)	1.17 (1.07, 1.29)	<0.001

* Matched on age, smoking status, date of blood drawing, and fasting status (women only)

† Values are least square mean ± standard error for continuous variables and proportions for categorical variables, except for CRP, which is shown as log-transformed geometric mean and 95% confidence interval

‡ P for difference between cases and controls by matching factor-adjusted t-Test for rows with means, and by Chi-Square Test for rows with proportions

§ Race unknown in 10 cases and 21 controls (HPFS); 18 cases and 16 controls (NHS)

**Table 2:** Genotype frequency for CRP polymorphisms among cases and controls in Nurses' Health Study (Women) and Health Professionals Follow-up Study (Men)

		Women			Men		
		Cases	Controls	p-value*	Cases	Controls	p-value*
**1919A>T**	rs1417938						
	AA	122 (51.7)	231 (49.6)		122 (50.0)	234 (48.1)	
	AT	93 (39.4)	185 (39.7)		98 (40.2)	201 (41.3)	
	TT	21 (8.9)	50 (10.7)	0.72	24 (9.8)	52 (10.7)	0.87
	Total	236	466		244	487	
							
**2667G>C**	rs1800947						
	GG	205 (85.1)	433 (89.8)		209 (81.0)	458 (89.3)	
	GC	35 (14.5)	49 (10.2)		45 (17.4)	51 (9.9)	
	CC	1 (0.41)	0 (0.0)	0.05	4 (1.6)	4 (0.8)	0.006
	Total	241	482		258	513	
							
**3014G>A**	rs1130864						
	GG	125 (52.7)	238 (51.0)		126 (50.8)	235 (47.8)	
	GA	94 (39.7)	178 (38.1)		98 (39.5)	205 (41.7)	
	AA	18 (7.6)	51 (10.9)	0.39	24 (9.7)	52 (10.6)	0.73
	Total	237	467		248	492	
							
**3872C>T**	rs1205						
	CC	107 (44.4)	210 (44.2)		111 (43.7)	237 (46.7)	
	CT	108 (44.8)	225 (47.4)		113 (44.5)	222 (43.7)	
	TT	26 (10.8)	40 (8.4)	0.54	30 (11.8)	49 (9.6)	0.57
	Total	241	475		254	508	
							
**4741G>C**	rs3093068						
	GG	206 (86.6)	419 (89.2)		233 (90.0)	435 (86.5)	
	GC	32 (13.5)	48 (10.2)		25 (9.6)	60 (11.9)	
	CC	0 (0.0)	3 (0.64)	0.22	1 (0.4)	8 (1.6)	0.22
	Total	238	470		259	503	
							
**5237A>G**	rs2808630						
	AA	126 (52.3)	216 (45.4)		124 (51.0)	256 (51.3)	
	AG	85 (35.3)	224 (47.1)		96 (39.5)	204 (40.9)	
	GG	30 (12.5)	36 (7.6)	0.004	23 (9.5)	39 (7.8)	0.74
	Total	241	476		243	499	

* P, chi-square, or Fisher's exact test for cells less than 20.

**Table 3:** Pairwise linkage disequilibrium (D') and correlation coefficients (r^2^) between CRP polymorphisms and control women and men

Women			Linkage Disequilibrium (D')				
	Polymorphism	1919	2667	3014	3872	4741	5237
Correlation Coefficient (r^2^)	1919	-	1.000	0.9838	0.9823	1.000	1.000
	2667	0.0235	-	1.000	0.9553	1.000	0.7563
	3014	0.9526	0.0237	-	0.9822	1.000	0.9611
	3872	0.2003	0.1034	0.2027	-	1.000	1.000
	4741	0.0278	0.0034	0.0260	0.0305	-	1.000
	5237	0.1945	0.0134	0.1729	0.2152	0.0269	-
Men	Polymorphism	1919	2667	3014	3872	4741	5237
Correlation Coefficient (r^2^)	1919	-	1.000	0.9950	0.9835	1.000	1.000
	2667	0.0273	-	1.000	0.9680	1.000	1.000
	3014	0.9900	0.0270	-	0.9517	0.8431	1.000
	3872	0.1988	0.1260	0.1912	-	0.7588	1.000
	4741	0.0362	0.0050	0.1038	0.0209	-	1.000
	5237	0.1845	0.0242	0.1820	0.1822	0.0335	-

### CRP SNPs, plasma CRP levels, and risk of coronary heart disease

Under the additive model, 2667G>C was associated with an increased risk of CHD in both women (RR 1.57, 95% confidence interval [CI] 1.01–2.44) and men (RR 1.93, 95% CI 1.30–2.88); however, none of the other SNPs were associated with risk of CHD in both men and women (**Table 4**). Multivariable adjustment did not change these results. Furthermore, among the controls, individual SNPs were associated with plasma CRP levels, with similar associations and directions for both women and men (**Table 4**). Under the same model, SNPs 2667G>C and 3872C>T were associated with 22–37% lower CRP levels, whereas 4741G>C was associated with ∼40% higher CRP levels. SNPs 1919A>T and 3014G>A were associated with approximately 20% higher CRP levels, whereas 5237A>G was not associated with CRP levels in either men or women.

**Table 4:** Association of CRP SNPs with risk of coronary heart disease, and with plasma CRP levels among women and men

	Women					Men				
	Cases/Controls	Odds ratio (95% CI)*	p-value	Multiplicative change in CRP levels†	p-value‡	Cases/Controls	Odds ratio (95% CI)*	p-value	Multiplicative change in CRP levels†	p-value‡
**1919A>T**	236/466	0.90 (0.70–1.14)	0.37	1.22	0.009	244/487	0.93 (0.73–1.18)	0.55	1.18	0.02
**2667G>C**	241/482	1.57 (1.01–2.44)	0.047	0.63	0.01	258/513	1.93 (1.30–2.88)	0.001	0.72	0.01
**3014G>A**	237/467	0.88 (0.69–1.12)	0.28	1.15	0.08	248/492	0.89 (0.70–1.13)	0.32	1.22	0.006
**3872C>T**	241/475	1.08 (0.85–1.37)	0.54	0.76	0.001	254/508	1.17 (0.92–1.48)	0.88	0.78	0.001
**4741G>C**	238/470	1.22 (0.76–1.94)	0.42	1.38	0.006	259/503	0.70 (0.44–1.09)	0.12	1.41	0.01
**5237A>G**	241/476	0.93 (0.72–1.19)	0.56	0.98	0.86	243/499	1.07 (0.84–1.37)	0.59	0.96	0.56

* Conditional logistic regression under the additive genetic model

† Matching factor-adjusted, log-transformed, multiplicative change per copy of the allele among controls

‡ P for change in CRP levels

### CRP haplotypes, plasma CRP levels, and risk of coronary heart disease

Five common haplotypes (H1-H5) had an estimated frequency ≥5% (**Table 5**). H1 with minor alleles of 1919A>T and 3014G>A and major alleles of all others, was the most frequent haplotype and set as the reference haplotype in subsequent analyses. H2, with minor allele of 5237A>G, and H3, with minor allele of 3872C>T, had estimated frequencies ranging from 25–30%. Neither H2 nor H3 was associated with risk of CHD. However, H4, with an estimated frequency of 6–7%, was associated with an increased risk of CHD in both women (RR 1.66, 95% CI 1.02–2.71) and men (RR 1.90, 95% CI 1.25–2.88). The difference between H3 and H4 may be attributable to the minor allele of 2667G>C.

**Table 5:** Common CRP haplotypes, risk of CHD, and relative effects of haplotypes on plasma CRP levels

CRP SNP	1919	2667	3014	3872	4741	5237				Multiplicative change in CRP per copy relative to H1 among controls‡	
*Women*	A>T	G>C	G>A	C>T	G>C	A>G	Freq (%)	OR (95%CI)†	p-value		p-value
Haplotype #:											
H1	1	0	1	0	0	0	29.1	1.0	-	Ref	-
H2	0	0	0	0	0	1	30.7	1.01 (0.77–1.34)	0.93	0.88	0.14
H3	0	0	0	1	0	0	26.4	0.99 (0.74–1.34)	0.97	0.76	0.006
H4	0	1	0	1	0	0	5.8	1.66 (1.02–2.71)	0.04	0.56	0.002
H5	0	0	0	0	1	0	6.0	1.27 (0.79–2.04)	0.32	1.19	0.15
H6§	-	-	-	-	-	-	<5.0	0.93 (0.39–2.24)	0.88	0.68	0.26
p-value¥	0.37										
CRP SNP	1919	2667	3014	3872	4741	5237				Multiplicative change in CRP per copy relative to H1 among controls‡	
*Men*	A>T	G>C	G>A	C>T	G>C	A>G	Freq (%)	OR (95%CI)†			p-value
Haplotype #:											
H1	1	0	1	0	0	0	30.8	1.0	-	Ref	-
H2	0	0	0	0	0	1	28.7	1.11 (0.84–1.47)	0.45	0.86	0.06
H3	0	0	0	1	0	0	24.8	1.03 (0.77–1.37)	0.87	0.76	0.004
H4	0	1	0	1	0	0	7.1	1.90 (1.25–2.88)	0.003	0.64	0.002
H5	0	0	0	0	1	0	6.7	0.82 (0.52–1.30)	0.40	1.19	0.27
H6§	-	-	-	-	-	-	<5.0	1.04 (0.45–2.39)	0.93	0.97	0.89
p-value¥	0.04										

* Cutoff set as ≥5.0%, based on 744 women and 781 men.

† Haplotype-specific ORs (multiplicative increase in odds per copy of haplotype) using unconditional logistic regression, adjusted for matching factors

‡ Log-transformed CRP adjusted for matching factors

§ All other haplotypes

¥ Globally-adjusted likelihood ratio test

Where individual SNPs were associated with CRP levels, estimated haplotypes were associated with plasma CRP levels in a similar manner. The tagging SNPs for H1 were individually associated with higher CRP levels. Compared to H1, H3 and H4 were associated with 24–44% lower CRP levels among both men and women, and these findings were as expected from their individual variant alleles. For comparison, H1 and H5, which included the triallelic proxy, were both associated with higher CRP levels.

Overall, the global p-value comparing the model with haplotypes to the model without was not statistically significant among women (p = 0.37) but significant among men (p = 0.04).

## Discussion

In two independent populations of predominantly Caucasian men and women, we related common variation in the CRP gene and estimated common haplotypes with plasma CRP levels and risk of incident CHD. CRP SNPs and associated haplotypes were strongly associated with plasma CRP levels. However, neither the individual SNPs nor common haplotypes were associated with risk of CHD in the direction that would be predicted by their association with CRP levels.

Inflammation is thought to contribute to increased cardiovascular risk, and C-reactive protein, an important component of the innate immune system, is the most extensively studied marker of inflammation.[3]–[6] Many studies have reported individual single nucleotide polymorphisms (SNPs) to be significantly associated with baseline CRP levels (**Table 6**).[9], [13]–[21], [23], [24], [36], [37] Though some of the individual SNP results have been conflicting, our results are consistent with the majority of published findings.

**Table 6:** Brief summary of literature examining single CRP SNPs with CRP levels, and CHD where available

	Association with CRP levels			Association with CHD		
	↑ levels	↓ levels	no association	↑ risk	↓ risk	no association
**1919A>T**	[14], [20]		[23], [36]			
**2667G>C**	[21]	[14]–[16], [20], [23]	[13], [18]			[15]
**3014G>A**	[13], [16], [19], [20], [22]		[18]			[19], [22]
**3872C>T**		[16], [20], [22], [23]				[22]
**4741G>C**	[22], [23], [37]					[22]
**5237A>G** *			[18], [20], [21], [23]		[23]	

* SNP 5237 was in high LD with another commonly reported SNP -717A>G (rs2794521).

On the other hand, the studies that assessed common variation and haplotypes in the CRP gene with plasma CRP levels differed in SNP selection methodology and estimated haplotypes.[9], [16], [22]–[24], [32], [37] For example, Carlson et al.[32] resequenced 47 samples from panels with African American and European descent individuals to select seven tagging SNPs for eight haplotypes in the CARDIA study. On the other hand, Miller et al.[23] resequenced 192 individuals in the Women's Health Study with extreme discordant baseline CRP levels and selected seven tagging SNPs for six common haplotypes among Caucasians participants in three sub-cohorts. In the Rotterdam Study, Kardys et al. genotyped 3 tagging SNPs (3014, 3872, and 4741) selected from SeattleSNPs which were sufficient to infer four common haplotypes.[22] Though overall haplotypes were not exactly replicated, the more recent studies shared much overlap in selected SNPs. Based on LD patterns and similar reference groups, our results were similar to what has been previously reported. The haplotypes associated with lower CRP levels were similar, as were the haplotypes associated with higher CRP levels.

Several studies have shown H4 as associated with significantly lower CRP levels.[9], [16], [23], [24], [32] However, specifically of importance, few studies have assessed haplotypes, and H4 in particular, with risk of incident CHD. Thus far, the results have been unclear and conflicting. Though the Rotterdam Study did not genotype 2667G>C, they reported four haplotypes significantly associated with CRP levels, and reported no associations with CHD.[22] A recent study in the Cardiovascular Health Study of older adults reported no association between five CRP haplotypes and risk of MI among white participants, and the relative risk of MI for our H4 equivalent was 0.93 (95% CI 0.72–1.21).[24] On the other hand, though H4 was not associated with CRP levels in the Physicians' Health Study, 3872C>T individually was associated with lower CRP levels, and with an increased risk of MI (odds ratio: 1.29, 95% CI 0.99–1.67).[23] Furthermore, the odds ratio for 2667G>C was suggestive of a 50% increase as well, but the confidence interval was much wider. Taken together, these results suggest that 2667G>C and 3872C>T, though associated with lower CRP levels, may be associated with an elevated risk of CHD.

A number of SNPs in the promoter region of the CRP gene have been shown to affect changes in transcription factor binding and gene promoter activity.[17], [32] The triallelic SNP has been associated with increased promoter activity and higher CRP levels,[32] and haplotypes of the promoter triallelic and rs3093032 SNPs affect transcription factor binding, transcriptional activity, and CRP levels.[17] The major alleles of 3872C>T and 5237A>G together serve as a proxy for the triallelic,[9] and the 2-SNP haplotype was associated with higher CRP levels in our studies as well as in others.[9], [23], [32] Otherwise, more functionality data are needed for CRP SNPs.

There is emerging consistency in the literature that polymorphisms in the CRP gene are associated with plasma CRP levels, but whether there is also an association between polymorphisms and risk of coronary events remains unclear. While CRP polymorphisms are strongly associated with plasma levels, these polymorphisms contribute only 1.4–5.0% of the phenotypic variation, a level comparable to (or less than) that from environmental and lifestyle factors.[9], [23] In addition to environmental stimuli, CRP is induced by other inflammatory cytokines, and additional genes in the inflammatory pathway may regulate CRP levels as well. Thus, our results are consistent with CRP as a good indicator of the inflammatory state and as a risk marker. However, the apparent influence in baseline CRP levels due to CRP polymorphisms may not be large enough to alter CHD risk, which may explain the lack of association between the majority of common haplotypes in the CRP gene and risk of CHD in either the NHS or the HPFS. The remaining exception would be haplotype 4, and it is possible that CRP is an epiphenomenon of vascular inflammation and not directly involved in vascular pathobiology, or possibly protective. Alternatively, the CRP polymorphisms and haplotypes may be in LD with pro-atherogenic variants in nearby gene regions which would not be captured in this study.

Several potential limitations should be discussed regarding the current two studies. In genetic association studies, population stratification is often a concern. However, population stratification should be minimized in the NHS and HPFS because both cohorts are predominantly Caucasian, and similar results were observed when restricted to Caucasians only. Additionally, we recognize that the relative socioeconomic homogeneity of the cohorts does not represent random samples of U.S. men and women and may not be generalizeable to other populations. Though the homogeneity is unlikely to influence genetic predisposition, it may be a strength in reducing residual confounding from unmeasured factors related to socioeconomic status. Also, the distribution of cardiovascular risk factors such as smoking status and history of diabetes were different between the men and women, and may have mediated influences through CRP. Finally, chance may be an explanation for the statistically significant findings. Nevertheless, we included a replication study by examining both the NHS and HPFS, and the overall results were identical between the two independent populations.

Our study provides a comprehensive assessment of the common genetic variation in the CRP gene with plasma CRP levels and risk of incident CHD among two independent populations. Among predominantly Caucasian men and women, CRP genotypes and haplotypes were consistently and significantly associated with plasma CRP levels. However, the relationship between genotype and CRP levels did not translate largely to an associated change in CHD risk, and Haplotype 4 in particular may be important in predicting other clinical events. These data suggest that the underlying inflammatory processes which predict coronary events cannot be captured solely by variation in the CRP gene, and other regulatory genes should be examined.
